# Distribution of *FMR1* and *FMR2* Repeats in Argentinean Patients with Primary Ovarian Insufficiency

**DOI:** 10.3390/genes8080194

**Published:** 2017-08-16

**Authors:** Lucía Daniela Espeche, Violeta Chiauzzi, Ianina Ferder, Mehrnoosh Arrar, Andrea Paula Solari, Carlos David Bruque, Marisol Delea, Susana Belli, Cecilia Soledad Fernández, Noemí Delia Buzzalino, Eduardo Hernán Charreau, Liliana Beatriz Dain

**Affiliations:** 1 Centro Nacional de Genética Médica-ANLIS, Avenida Las Heras 2670 3er piso, Ciudad Autónoma de Buenos Aires C1425ASQ, Argentina; lespeche@anlis.gov.ar (L.D.E.); tutandre@hotmail.com (A.P.S.); bruquecarlos@gmail.com (C.D.B.); marisoldelea@gmail.com (M.D.); cecisolfer@gmail.com (C.S.F.); nbuzzalino@gmail.com (N.D.B.); 2 Instituto de Biología y Medicina Experimental (IByME)-CONICET, Vuelta de Obligado 2490 Ciudad Autónoma de Buenos Aires C1428ADN, Argentina; vchiauzzi@ibyme.conicet.gov.ar (V.C.); ianinaf@gmail.com (I.F.); echarreau@gmail.com (E.H.C.); 3 Instituto de Química-Física de los Materiales, Medio Ambiente y Energía, CONICET, and Departamento de Química Inorgánica, Analítica y Química Física, Facultad de Ciencias Exactas y Naturales, Universidad de Buenos Aires, Ciudad Autónoma de Buenos Aires C1428EGA, Argentina; mehrnoosh.arrar@gmail.com; 4 División de Endocrinología, Hospital de Agudos Carlos G. Durand. Avenida Díaz Vélez 5044, Ciudad Autónoma de Buenos Aires C1405DCS, Argentina; susanabelli486@gmail.com

**Keywords:** primary ovarian insufficiency, *FXPOI*, *FMR1* premutation, *FMR2* microdeletions, premature menopause

## Abstract

The premutation state of *FMR1* (Fragile X Mental Retardation 1) has been associated with primary ovarian insufficiency (POI), and is the most common known genetic cause for 46,XX patients. Nevertheless, very few studies have analyzed its frequency in Latin American populations. Additionally, a relationship between alleles carrying a cryptic microdeletion in the 5’UTR of *FMR2* and the onset of POI has only been studied in one population. Our aim was to analyze the incidence of *FMR1* premutations and putative microdeletions in exon 1 of *FMR2* in a cohort of Argentinean women with POI. We studied 133 patients and 84 controls. Fluorescent PCR was performed, and the *FMR2* exon 1 was further sequenced in samples presenting less than 11 repeats. We found the frequency of *FMR1* premutations to be 6.7% and 2.9% for familial and sporadic patients, respectively. Among controls, 1/84 women presented a premutation. In addition, although we did not find microdeletions in *FMR2*, we observed a change (T >C) adjacent to the repeats in two sisters with POI. Given the repetitive nature of the sequence involved, we could not ascertain whether this represents a single nucleotide polymorphism (SNP) or a deletion. Therefore, a relationship between *FMR2* and POI could not be established for our population.

## 1. Introduction

Primary ovarian insufficiency (POI, formerly known as premature ovarian failure) is a syndrome clinically defined by ovarian failure before the age of 40. It is characterized by primary or secondary amenorrhea, hypoestrogenism, and elevated gonadotropin serum levels [1]. This syndrome is very heterogeneous with a multicausal pathogenesis: chromosomal, enzymatic, iatrogenic, autoimmune, or infectious [2]. The incidence of POI among women of reproductive age is estimated at 1% [3]. Because of its frequent association with a positive family history, POI was suggested to be a genetic disorder [4,5]. In some individuals, POI may occur as part of a complex phenotype involving tissues other than ovaries (syndromic POI). In most patients, however, POI occurs in an isolated manner (non-syndromic). Even though multiple candidate genes have been described in non-syndromic POI, the etiology of the condition remains unclear in up to 90% of the patients [6,7].

One of the most studied candidate genes is *FMR1* (Fragile X Mental Retardation 1, OMIM 309550). *FMR1* is located in Xq27 (FRAXA locus) and presents a CGG repeat in the 5’ untranslated region (UTR) in exon 1 that is unstable and therefore variable in the population. Based on the size of the repeat tract, individuals are classified as having normal (5–44 trinucleotide repeats), gray zone (45–54 repeats), premutation (55–200 repeats), or full mutation (>200 repeats) size alleles [8,9,10]. The presence of a full-mutation allele is responsible for the Fragile X syndrome—the most common known cause of inherited intellectual and developmental disability [11]. Additionally, the premutation state has been associated with two clinical disorders: the Fragile X-associated tremor/ataxia syndrome (FXTAS, a late-onset neurodegenerative disorder), and Fragile X-related primary ovarian insufficiency (FXPOI) [12,13,14,15].

FXPOI is the most common known genetic cause for 46,XX POI, and the premutation condition is responsible for 4–6% of these cases. About 2% of 46,XX women with sporadic POI and 14% of 46,XX women with familial POI carry a premutation in the *FMR1* gene [15]. Moreover, it has also been described that around 20% of women carrying a premutation experience menopause approximately 5 years earlier than do non-carriers [16,17]. The premutation status seems to influence the expression of the disease. In fact, no woman with POI with a full-mutation has been described.

The FRAXE locus—located in Xq28—has been associated with X-linked intellectual disability (OMIM 309548) affecting 1 of 50,000 newborn males [18]. This locus contains the *FMR2* gene (OMIM 300806, also known as *AFF2*), which encodes a putative transcriptional activator factor of the *AF4/FMR2* gene family. Similar to *FMR1*, its 5’UTR contains a polymorphic uninterrupted GCC trinucleotide repeat in exon 1 (dbSNP: rs193922937) [19,20]. In the general population, the number of this repeat varies between 6 and 35 [19,21], whereas alleles of more than 200 hypermethylated copies have been observed in intellectually disabled patients. This repeat tract can either expand or contract, and is equally unstable when transmitted through the male or the female germ line [19,22,23].

Aside from the proven relationship between *FMR2* and intellectual disability, alleles carrying a cryptic microdeletion (either at or near the transcription initiation site) in *FMR2* have been related to the onset of POI in two studies from the same population [24,25]. Nevertheless, no further studies analyzing this putative relationship in different populations have been carried out since.

The aim of the current study was to analyze the incidence of *FMR1* premutation alleles and the putative presence of microdeletions in exon 1 of the *FMR2* gene in a cohort of Argentinean women affected with POI. The frequency of *FMR1* premutations was in accordance with previous reports in different populations. In addition, we did not find microdeletions in *FMR2* in our sample. However, we observed a putative change (T >C) adjacent to the repeats in two sisters with POI but we were unable to ascertain whether this represented a single nucleotide polymorphism (SNP) or a deletion.

## 2. Materials and Methods

### 2.1. Ethical Approval

All clinical investigations were conducted according to the principles expressed in the Declaration of Helsinki. Written informed consent was obtained from all patients and controls involved in this study. The study was approved by the ethics committees from the Instituto de Biología y Medicina Experimental (CE 031-2/2011), and from the Centro Nacional de Genética Médica, Buenos Aires, Argentina (CE Acta #10, pg.38).

### 2.2. Patients and Controls

Patients were included from health institutions from the city of Buenos Aires and its surroundings. Inclusion criteria were previously established in our laboratory [26,27], and involved: having amenorrhea for over a year starting before the age of 40, serum follicle stimulating hormone (FSH) level above 40 mIU/mL (normal follicular phase levels: 2–9 mIU/mL) in two consecutive determinations and plasma 17β−estradiol levels below 15 pg/mL (normal follicular phase levels: 20–120 pg/mL). Patients presenting anti-follicle stimulating hormone receptor (FSHR) antibodies, determined by radioreceptor assay [28], as well as chromosomal abnormalities, were excluded from the present work.

The population studied comprised 133 patients with POI with 46,XX karyotype. The patients were considered idiopathic because they did not show any other POI-related condition (i.e., ovarian surgery, previous chemo- or radiotherapy, or metabolic disorders such as galactosemia). They experienced the insufficiency between 14 and 39 years old. Twenty-two patients presented primary amenorrhea, and 111 presented secondary amenorrhea.

Thirty patients from 25 families had a family history of premature menopause. From five families, we recruited two members: three pairs of twin sisters, one pair of sisters, and a mother and her daughter, whereas from the remaining 25 families, one of the affected members was included. One hundred and three patients were classified as having sporadic POI.

In addition, 84 women from the city of Buenos Aires and its surroundings above the age of 40 (4 pairs of sisters and a mother and her daughter) with normal menstrual history and without premature menopause were recruited voluntarily as controls [26,27]. All but three were of proven fertility, and had delivered at least one child. They all stated not having a familial history of POI nor intellectual disability.

The study of the repeat length in *FMR1* was performed for all the patients and controls, whereas the analysis of exon 1 of *FMR2* was done for 127 patients and all controls. The distribution analysis was assessed including only the non-related alleles. 

### 2.3. PCR and Sequencing

The PCR reactions were performed on DNA extracted from peripheral white blood cells with the “salting out” technique [29]. The 5’UTR region of *FMR1* was amplified by PCR with primers c and f [8]. *FMR2* exon 1 was amplified using the primers U35072 and 598, as described by Murray et al. [24]. The reverse primer of each PCR was FAM-labeled. The PCRs were performed in 50 μL final volume using approximately 300 ng of genomic DNA, 0.875 μL Pfx Taq (Pfx Taq Platinum polymerase, Invitrogen, Carlsbad, CA, USA), 1X Buffer (Platinum kit), 0.9 mM MgSO_4_ (Platinum kit), 0.125 mM dNTPs, 0.45 μM of each primer, 2X PCR Enhancer (Platinum kit), and 0.96 M Betaine solution (Sigma Aldrich, St. Louis, MO, USA). Cycling conditions were: 95 °C denaturation for 3 min followed by 32 cycles of 95 °C denaturation for 15 s, 62 °C annealing for 2 min, 68 °C elongation for 2 min, and a final elongation of 10 min at 68 °C.

The length of the *FMR1* fragments was determined based on their molecular weight on an Applied Biosystems 3500 genetic analyzer, with control samples with different CGG size alleles included in each run. These control samples (Coriell Biorepository, Camden, NJ, USA) held 23/30, 31/46, 31/53, and 30/73 repeats. The *FMR2* PCR was validated by sequencing two samples: a male subject with 15 repeats and a female with 17/21 repeats. These samples were further used as controls for the estimation of the triplet tract length in every PCR reaction and subsequent electrophoresis. Samples with 11 GCC or fewer were re-amplified with unlabeled primers and further resolved in a 3% agarose gel, and the corresponding band was excised and purified for Sanger sequencing. Nucleotide numbering was performed following the guidelines of the Human Genome Variation Society [30], using NG_016313.1 as the genomic *Homo sapiens* AF4/FMR2 family member 2 (*AFF2*), RefSeqGene on chromosome X reference sequence. The nucleotide sequence data reported is available in the GenBank database under the accession number KY689834.

### 2.4. Southern Blot

When sufficient DNA was available (11/42 samples), Southern blot was performed to exclude a full mutation size *FMR1* allele in the cases of putative homozygous status after fluorescence-PCR. For the remaining patients, homozygosity was assumed, considering that the number of repeats accurately detected by PCR in our laboratory was up to 190 triplets. Homozygosity was also assumed for the 21 patients that showed only one peak in the *FMR2* PCR.

Briefly, 10 µg of genomic DNA were double-digested with EagI and EcoRI (20U EagI, 30U EcoRI, 1X EagI Buffer (Thermo Scientific, Waltham, MA, USA), 0.02 mM spermidine, 37 °C and overnight incubation) and electrophoresed on a 1% agarose gel. The gel was then transferred to a nylon membrane and hybridized overnight at 42 °C with the digoxigenin-labeled probe pFXa1NHE (Chemicon International, Temecula, CA, USA) using DIG Easy Hyb solution (Roche Diagnostics, Mannheim, Germany). After hybridization, the fragments were visualized by immuno-chemiluminescence using DIG Wash and Block Buffer Set, anti-digoxigenin-Ap Fab Fragments, and CDP-Star (Roche Diagnostics, Mannheim, Germany) according to the manufacturer’s instructions.

### 2.5. Statistical Analyses

The exact two-tailed binomial test as implemented in the RStudio suite [31,32] was used to determine whether the observed incidence of patients with premutation size alleles was significantly different from previously reported literature values. For all case and control groups, we report the observed incidences as exact 95% confidence intervals for a binomially distributed variable, using the method described by Clopper and Pearson [33].

## 3. Results

We analyzed the distribution of CGG and GCC repeats in the 5’UTR region of *FMR1* and *FMR2* genes, respectively, in a group of patients affected with POI and in a group of controls.

### 3.1. Distribution and Allele Frequencies of FMR1 Repeats

**Figure 1:**
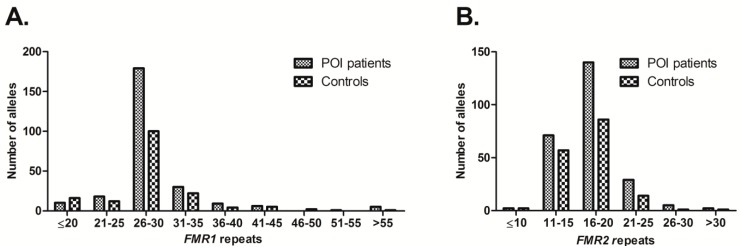
Distribution of the Fragile X Mental Retardation 1 (*FMR1)* and *FMR2* triplet repeats in primary ovarian insufficiency (POI) patients and controls; (**A**) Repeat distribution for the *FMR1* gene. The modal number of CGG repeats was 30 (patients 91/258, controls 64/162), followed by 29 (patients 83/258, controls 35/162); (**B**) Repeat distribution for the *FMR2* gene. The most frequent number of repeats was 15 among patients (53/245) as well as among controls (51/161), followed by 18 (patients 50/245, controls 29/161). Only non-related alleles were considered.

The observed range for the repeats distribution was 19–109 for patients and 12–79 for controls (Figure 1). The modal number of CGG repeats in both patients and controls was 30, followed by 29.

The size range of premutation alleles in POI patients was 63–109. In one sporadic patient, the exact number of repeats (54 or 55) could not be determined, and therefore was not included as a premutation. Five patients with POI had a premutation in one of their alleles (Table 1), two of them being mother and daughter (familial POI), which resulted in an estimated frequency of 6.7% for familial cases (2 out of 30) and 2.9% for sporadic cases (3 out of 103 patients). In the familial case where a premutation was found, the mother had an allele of 85 repeats that expanded to 104 when transmitted to her daughter. Four out of the five patients with a premutation carried an allele with repeat size over 100. In the control group, 1 out of 84 women presented a premutation (79 CGG repeats). Taking into account that the prevalence of *FMR1* premutation carriers was reported in other population-based studies as 3.3 in 1000 [34], it would appear as though the premutation is three-fold more prevalent in the control population in this study; however the 95% confidence interval of the true probability of premutation in our control group (0.03 to 6.45%) includes the previously reported probability of 3.3 in 1000 (*p*-value 0.2).

None of the samples analyzed in the control or the patient groups revealed an allele with a full mutation.

It has also been proposed that intermediate *FMR1* alleles could be associated with POI development [35,36]. We analyzed the number of POI patients and controls with intermediate alleles using a range of 35–54 repeats (as proposed by Bretherick et al. [35]), as well as a range of 41–58 repeats (as proposed by Bodega et al. [36]). As shown in Table 1, the distribution of intermediate alleles was similar between patients and controls.

### 3.2. Distribution and Allele Frequencies of FMR2 Repeats and Sequencing

**Figure 2:**
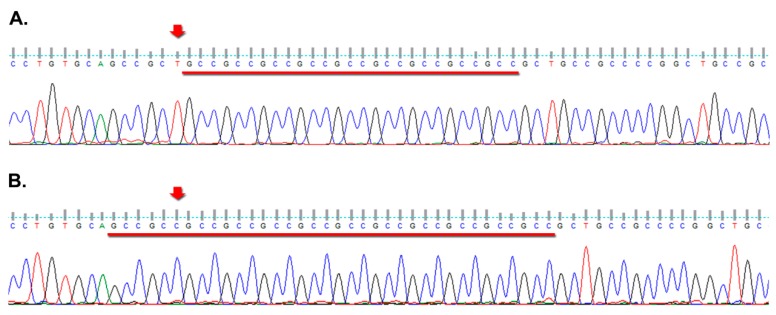
Electropherogram images from two *FMR2* exon 1 forward sequencing reactions; (**A**) The arrow points the T nucleotide present in the reference sequence (NG_016313.1); (**B**) Illustrates the sequence from one of the twin sisters with POI, with the arrow pointing the position where a T was expected. Underlined is the GCC tract.

The most frequent number of repeats for *FMR2* was 15 among patients (range: 10–33) as well as among controls (range: 9–37), followed by 18 in both groups (Figure 1). Our results also revealed that only one sporadic patient presented 10 repeats. In addition, two controls had an allele with 9 and one had an allele with 11 repeats (Table 2). None of them presented a deletion in the analyzed region. Additionally, two monozygotic twins in the POI sample group presented an allele with apparently 10 repeats in the PCR. After direct sequencing, we found a change adjacent to the repeats (Figure 2). Given the repetitive nature of the sequence involved, we could not ascertain whether the T preceding the repeats (nucleotide position 5019 from the NG_016313.1 *AFF2* sequence) was either changed for a C or represents a deletion, leaving a (GCC)_12_ tract.

All patients and controls who presented “minimum alleles” in *FMR2* had a normal number of repeats in *FMR1*.

## 4. Discussion

Despite the evidence that the premutation in *FMR1* is associated with POI [14,15,17,37,38,39], there are very few studies that analyzed this relationship in Latin American populations [40,41]. Moreover, none of them have focused on the prevalence of the premutation in women with POI; rather, they studied the occurrence of POI in women with a premutation from Fragile X families.

The modal numbers of *FMR1* repeats observed in our cohort were in accordance with those reported in Brazil [42,43] and in other populations of European ancestry [8,14,44,45,46,47,48,49,50]. Similarly, we found a prevalence of premutations among POI patients that is in accordance with previous reports that have described a frequency ranging from 0.8% to 7.5% in women with sporadic POI and up to 14%, with an overall incidence of around 4% [51], in women with familial POI [15,24,38,39,52]. In addition, although the prevalence of *FMR1* premutation carriers was reported in other population-based studies as 3.3 in 1000 females from the general population [34], our result of 1 carrier in 84 women is not inconsistent with the reported prevalence, as the 95% CI includes the reported value, thus warranting further studies with larger sample sizes.

Some groups have described an increased probability of POI with increasing number of CGG repeats for *FMR1*. However, this risk appears to be nonlinear, as it increases with the premutation size but it plateaus or is even reduced in women with repeat sizes over 100 [17,53]. In our sample, most of the premutation patients carried an allele with more than 100 repeats. Nevertheless, more patients carrying a premutation would need to be studied to assess this relationship in our population.

Although Southern Blot was not performed for all apparently homozygous samples, our PCR method amplifies alleles up to 190 repeats for *FMR1* (data not shown). Moreover, it has not been published that a full mutation allele for *FMR1* or a high allele for *FMR2* was associated to POI [54]. When focusing on the intermediate CGG range, Bretherick et al. [35] observed a significant increase in the number of alleles with ≥35–54 repeats in a group of patients with POI. Similarly, Bodega et al. [36] found a significant enrichment of expansions in the range of 41–58 repeats in POI carriers. In contrast, Bennett et al. [48] found no significant difference between the incidence of intermediate alleles (35–58 repeats) in POI cases and that observed in controls. Moreover, Murray et al. [55] also studied women with intermediate alleles (45–54 repeats), and found that these alleles were not significant risk factors for either early menopause or POI. Considering these conflicting results, we analyzed the number of POI patients and controls with alleles included in the two proposed CGG size ranges. In accordance with the observations made by Bennett et al. and Murray et al. [48,55], we found that the 95% confidence intervals of the frequency of intermediate alleles include that of the control group for both the CGG size ranges considered.

Conversely to the extensively proven relationship between *FMR1* premutations and the risk for developing POI, the association between microdeletions in *FMR2* and the onset of the disease has not been widely evaluated. Only two studies from the same population have intended to analyze the relationship between POI and the FRAXE locus [24,25]. Specifically, an excess of alleles with fewer than 11 repeats—the so-called “minimum alleles”—was found in a cohort of 209 women with POI. This excess was later attributed to 3 out of 6 POI patients carrying cryptic deletions, in contrast to 1 out of 16 control individuals, thus leading to the proposal that microdeletions within *FMR2* may be a significant cause of POI [25]. Therefore, we analyzed the distribution of the *FMR2* repeats in our cohort and sequenced the samples having 11 or fewer repeats to avoid missing a microdeletion due to a possible small bias in the repeat count.

The two most frequently observed numbers of *FMR2* triplet repeats found in individuals from our population were similar to those previously described in samples from Euro-American, Finnish, and Chinese populations [20]. In addition, we found a sequence change in two sisters that could not be classified as either a SNP or a deletion for the repetitive nature of the sequence involved. However, the change does not fall in the transcription start site or in a known enhancer sequence. Thus, its relevance could not be assessed. Besides, we did not find cryptic deletions in exon 1 of the *FMR2* gene in the other samples analyzed; therefore, a relationship with the occurrence of POI could not be ascertained. Notably, all the patients carrying cryptic deletions described by Murray et al. [25] had apparently six or fewer repeats in the PCR, whereas the lowest number of repeats in the individuals from our cohort was nine GCC.

## 5. Conclusions

Our report represents one of the few studies that contribute to a better knowledge of the distribution of these alleles in Latin American populations. We found an estimated frequency of *FMR1* premutations of 6.7% and 2.9% for familial and sporadic POI cases, respectively. Even though a larger sample could provide a more accurate incidence, our results are in accordance with the observed frequencies of premutation among POI patients in different populations. In the control group, 1 out of 84 women presented a premutation. In addition, although alleles carrying a cryptic microdeletion in *FMR2* have been related to the onset of POI in studies from one population, no further reports analyzing this putative relationship have been carried out since. We found a sequence change in two sisters that could not be classified as either an SNP or a deletion due to the repetitive nature of the sequence involved; thus, its relevance could not be assessed. Considering that we did not find cryptic deletions in exon 1 of the *FMR2* gene in the other samples analyzed, a relationship with the occurrence of POI could not be ascertained. We believe that further studies should be performed in our population as well as in others to have a better understanding of the possible pathogenic involvement of *FMR2* in non-syndromic POI.

## Figures and Tables

**Table 1:** Percentage of primary ovarian insufficiency (POI) patients and controls with intermediate- and premutation-size Fragile X Mental Retardation 1 (*FMR1*) alleles.

Range of Intermediate and Premutation Alleles	Percentage (95% CI) of POI Patients (*n* = 133)		Percentage (95% CI) of Controls (*n* = 84)
	Sporadic (*n* = 103)	Familial (*n* = 30)	
35–54	* 12.6% (7–21%)	3.3% (0–5.3%)	14.3% (7.6–23%)
41–58	5.8% (2–12%)	0.0% (0–4.0%)	8.3% (3.4–16%)
58–109	2.9% (0.8–8.3%)	6.7% (0.24–6.8%)	1.2% (0.03–6.4%)

* One sporadic patient has a 38/43 genotype. CI: Confidence interval.

**Table 2:** Percentage of minimum *FMR2* alleles in POI patients and controls, after PCR.

*FMR2* Minimum Alleles	Percentage (95%CI) of POI Patients (*n* = 133)		Percentage (95%CI) of Controls (*n* = 84)
	Sporadic (*n* = 103)	Familial (*n* = 30)	
9	0.0% (0–3.5%)	0.0% (0.0–11.6%)	2.4% (0.29–8.3%)
10	1.0% (0.02–5.3%)	6.7% (0.81–22.1%)	0.0% (0.0–4.3%)
11	0.0% (0–3.5%)	0.0% (0.0–11.6%)	1.2% (0.03–6.5%)
